# Iron Homeostasis in *Azotobacter vinelandii*

**DOI:** 10.3390/biology12111423

**Published:** 2023-11-12

**Authors:** Elena Rosa-Núñez, Carlos Echavarri-Erasun, Alejandro M. Armas, Viviana Escudero, César Poza-Carrión, Luis M. Rubio, Manuel González-Guerrero

**Affiliations:** 1 Centro de Biotecnología y Genómica de Plantas (UPM-INIA/CSIC), Campus de Montegancedo UPM, Crta. M-40 km 38, 28223 Madrid, Spain; elena.rosa@upm.es (E.R.-N.); carlos.echavarri@upm.es (C.E.-E.); alejandro.armas@upm.es (A.M.A.); cpoza@cnb.csic.es (C.P.-C.); luis.rubio@csic.es (L.M.R.); 2 Escuela Técnica de Ingeniería Agraria, Alimentaria, y de Biosistemas, Universidad Politécnica de Madrid, Avda. Puerta de Hierro, 2, 28040 Madrid, Spain

**Keywords:** biological nitrogen fixation, nitrogenase, iron–sulfur cluster, iron transport, iron nutrition

## Abstract

**Simple Summary:**

*Azotobacter vinelandii* is a model organism used to study biological nitrogen fixation, a process by which nitrogen gas is transformed into ammonia, a form of nitrogen that can be assimilated by most organisms. This requires the synthesis and transfer of specific iron-containing cofactors to enzymes involved in nitrogen fixation. There are large gaps in our knowledge of iron uptake and utilization by *A. vinelandii*. In this review, our goal is to summarize current knowledge, propose novel elements based on our current understanding of bacterial iron homeostasis, and highlight those areas requiring more detailed research.

**Abstract:**

Iron is an essential nutrient for all life forms. Specialized mechanisms exist in bacteria to ensure iron uptake and its delivery to key enzymes within the cell, while preventing toxicity. Iron uptake and exchange networks must adapt to the different environmental conditions, particularly those that require the biosynthesis of multiple iron proteins, such as nitrogen fixation. In this review, we outline the mechanisms that the model diazotrophic bacterium *Azotobacter vinelandii* uses to ensure iron nutrition and how it adapts Fe metabolism to diazotrophic growth.

## 1. Introduction

Iron is an essential nutrient for life. It is a critical cofactor in numerous enzymes that use it as cofactor, either alone, as a part of heme groups or in iron–sulfur (Fe-S) clusters [1,2]. Typically, the role of iron in biological systems is based on its capacity to oscillate between Fe^2+^ and Fe^3+^ under physiological conditions. However, although iron is required at relatively low concentrations, at slightly higher concentrations, iron becomes toxic as it can displace other metals from the active site of enzymes or non-enzymatically catalyze the production of free radicals in Fenton reactions [3,4,5]. As a result, organisms must ensure a steady supply of iron to sustain key physiological processes and keep it under tight control to prevent iron toxicity. While every organism strives to maintain iron homeostasis, soil bacteria, especially those living in the rhizosphere (the soil area influenced by plant root exudates), have the added challenge of adapting to a heterogeneous soil composition. Iron availability is severely limited in most soils, as iron precipitates and becomes inaccessible [6]. This results in a tug-of-war for iron uptake between the different soil organisms, with important implications for plant nutrition and health and for the overall structure of plant-associated microbial communities [7,8,9]. 

To adapt and thrive under these conditions, bacteria devote considerable resources to the production, secretion, and recovery of siderophores, the synthesis and energization of specific transporters, the directional transfer of iron to the plethora of iron proteins in a cell, and the storage and detoxification of excess iron [10]. All of these are tightly regulated at the transcriptional, post-translational, and kinetic levels [11,12,13]. While a number of studies have described the mechanisms governing iron homeostasis in various bacteria [14,15,16], no recent study has focused on free-living diazotrophic microorganisms. This is despite the large iron requirements of nitrogen fixation [17], the only biochemical process that can convert dinitrogen (N_2_) to ammonia [18]. 

In this review, we will present what is known about the iron management by the model diazotroph *Azotobacter vinelandii*. We will list the genes that have a known or predicted role in iron homeostasis in this organism, and infer from related bacterial systems how effective iron nutrition and utilization can be achieved. The focus on *A. vinelandii* is based on its importance as a model to study the genetics and biochemistry of nitrogenase and in its agronomical and environmental relevance. *Azotobacter* is globally distributed, contributing to nitrogen fixation in a diverse range of conditions [19]. This is facilitated by *A. vinelandii* that synthesizes the three known types of nitrogenase: the iron–molybdenum nitrogenase, the iron–vanadium nitrogenase, and the iron-only nitrogenase [17]. This makes *A. vinelandii* ideal not only to study the role of the different nitrogenases but also their regulation by metal cofactor availability. Moreover, *A. vinelandii* is an excellent model to study branched electron transport chains, in addition to providing energy to metabolic reactions, it also protects nitrogenase against O_2_ [20]. Studying these mechanisms of nitrogenase protection is particularly important towards engineering nitrogen fixation in eukaryotes [21]. When colonizing the plant rhizosphere, *A. vinelandii* improves plant growth and nutritional value not only through nitrogen fixation but also by releasing hormones such as indole acetic acid [22,23]. This positive effect on plant growth can also be attributed to an increased tolerance to biotic stresses, as it has been shown that *A. vinelandii* could also act as a biocontrol agent [24].

## 2. Iron Uptake from Environment

**Figure 1:**
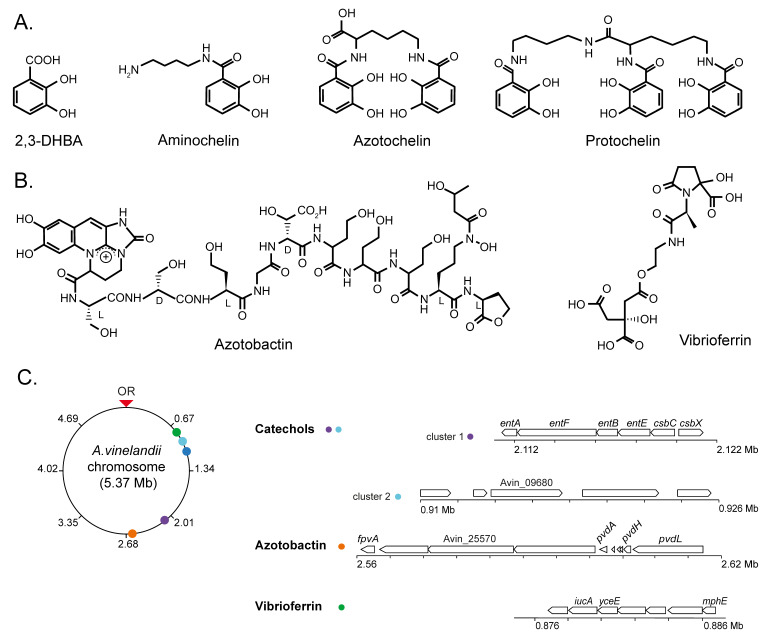
Representative siderophores secreted by *A. vinelandii* cultures. (**A**) Catechol-based siderophores: 2,3-DHBA, aminochelin, azotochelin, and protochelin. (**B**) Mixed-type siderophores: azotobactin and vibrioferrin. (**C**) Chromosome position and organization of the *A. vinelandii* DJ (genome accession NC_012560) gene clusters involved in siderophore production. OR indicates the origin of replication.

In spite of the iron abundance in the Earth’s crust (being the fourth most abundant element) [25], iron bioavailability is often limited by its low solubility at neutral-to-high pH, its conversion to iron oxides, and/or its retention by negatively charged particles in soils [26,27]. Therefore, most soil organisms secrete a wide range of siderophores, molecules that can bind iron with a high affinity and make it accessible to the bacteria [28,29]. Considering the wide ecological distribution of *A. vinelandii*, it is not surprising that it can secrete multiple molecules with different chemical structures. These molecules can be classified in two categories: catechols (in which iron coordination is performed by hydroxyl groups) and mixed types (in which iron coordination is performed by carboxylates and hydroxyls). Among the catechols, the most abundant ones are aminochelin, azotochelin, protochelin, and 2,3 DBHA (Figure 1A), while azotobactin and vibrioferrin are the main representatives of the mixed types (Figure 1B) [30,31,32]. These different siderophores can be further modified to alter metal-binding affinities and in some cases also metal selectivity [31,33]. This diversity of secreted siderophores may be a consequence of adaptation to different physiological and environmental stresses, related to the versatility of *A. vinelandii* in colonizing a wide range of environments [34]. Each siderophore has different chemical properties (such as solubility or iron-binding affinities) that may be useful for its adaptation to changing conditions. For instance, azotobactins are produced at higher levels in severely low iron conditions (0.1 μM Fe + 100 μM EDTA), whereas they are barely detectable at less-deprived ones (such as 5 μM Fe + 100 μM EDTA) [31,35]. Alternatively, the various secreted siderophores can be organized as “bucket brigades” to deliver iron to the host. This has been proposed for the coordinated action of vibriobactin and azoto/amino-chelins [31]. A highly hydrophilic vibriobactin, produced at high levels, but with a relatively low metal affinity, may act as a wide net to capture iron that could then be transferred to azotochelins or aminochelins that are more hydrophobic but with a higher iron affinity, which would then be introduced into the cell. Alternatively, the high diversity of siderophores could be caused by the ability of soil microorganisms to use xenosiderophores, siderophores synthesized by other organisms, to satisfy their own iron demands [36]. Having a wide range of siderophores could be a means of minimizing some of the losses to other organisms, and even sequestering a larger pool of iron, thus reducing competitors. Finally, siderophores might also provide protection against the toxic levels of transition metals or those that are not bioelements (such as cadmium or mercury) [37].

**Figure 2:**
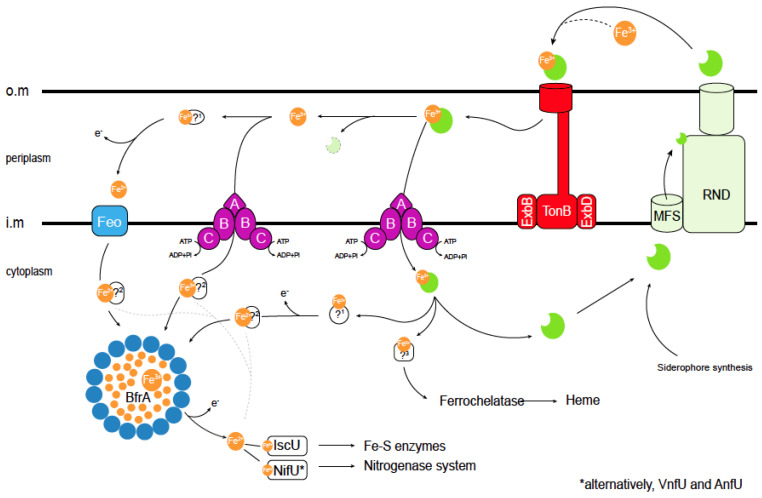
Current understanding of iron acquisition and trafficking in *A. vinelandii* cells. Siderophores secreted into the environment bind Fe^3+^. The resulting complex is introduced in the periplasm through TonB transporters. In the periplasm, different pathways can be followed: (i) the Fe^3+^–siderophore complex is transported through an ABC system into the cytosol, (ii) Fe^3+^ dissociates or is released from the siderophore and then it is transported into the cytosol by a different ABC transporter, or (iii) dissociated Fe^3+^ is reduced by an unknown ferroreductase (1) into Fe^2+^, which is transported by a Feo iron import system into the cytosol. Cytosolic Fe^2+^ or Fe^3+^ can be delivered directly to Fe-S scaffold proteins or to other iron-using enzymes (dotted lines). Alternatively, the excess iron can be stored within (bacterio) ferritins, and may later be mobilized after reducing Fe^3+^ to Fe^2+^ including siderophore biosynthesis. Iron trafficking in the cytosol is facilitated by yet-to-be-identified iron chaperones (2). As in the periplasm, iron is also recovered from the internalized Fe^3+^–siderophore complex, and Fe^3+^ may be reduced to Fe^2+^. Finally, an unknown protein (3) will be responsible for iron delivery to the ferrochelatase for heme synthesis. Siderophores are shown in green. Bacterioferritin (BfrA) protein subunits are shown as blue circles. TonB stands for (phage) *T-on*e resistance B; ExbB/D, for *Ex*cretion of colicin *B* inhibitor B/D; MFS is *M*ajor *F*acilitator Superfamily; RND, *R*esistance-*N*odulation-*D*ivision; Feo, *fe*rr*o*us iron transport; IscU, *I*ron *S*ulfur-*C*luster assembly U; NifU, *Ni*trogen *Fi*xation U; VnfU, *V*anadium *n*itrogen *f*ixation U; and AnfU, *A*lternative *n*itrogen *f*ixation U (iron-only nitrogenase). O.m: outer membrane; i.m. cytosolic membrane; and ?: proteins that have not been identified to date. * indicates that VnfU or AnfU are used instead of NifU for alternative nitrogenases.

The secretion of these siderophores is carried out by specific transporters (Figure 2), typically of the Major Facilitator Superfamily (MFS) or the Resistance-Nodulation-Division (RND) families that are encoded by siderophore biosynthesis gene clusters [38,39]. The MFS transporter CsbX, encoded by a catechol siderophore biosynthesis operon (Figure 1C), is up-regulated by an iron deficiency, consistent with a role in iron uptake [40]. As expected, the mutation of *csbX* results in the loss of catechol efflux capabilities. Transport across the outer membrane would typically be mediated by RND proteins. Although no specific protein has been identified for *A. vinelandii*, it has been shown that *Pseudomonas aeruginosa* releases pyoverdine (also synthesized by *A. vinelandii*), an intermediary in azotobactin biosynthesis [31] via the OprK mexAB system [41,42]. 

Iron siderophores must be recovered from soil, a task that is mediated by TonB-dependent receptors in the outer membrane [43,44] and ATP-Binding Cassette (ABC) transporters in the inner one (Figure 1C and Figure 2) [45]. The gene encoding the PsuA TonB-like protein is included in the vibrioferrin operon in *A. vinelandii* (Figure 2), whereas others predicted to encode iron-chelate transporters are found elsewhere in the genome. TonB proteins release their substrate in the periplasm, where typically the A subunit of an ABC transporter would mediate iron-chelate uptake into the cytosol through a dimer of the B subunit. Energy is provided by the ATPase encoded by the C subunit (Figure 2) [46]. This role in iron-catechol uptake is supported by the presence of genes encoding an ABC transporter in the catechol siderophore operon (Figure 1C).

However, iron-chelates are not the only chemical species of transported iron. Fe^2+^ transporters of the FeoB family are encoded in the *A. vinelandii* genome. These are GTP-gated permeases that facilitate iron transport across the inner membrane (Figure 2) [47]. Typically, they are encoded by genes that form an operon that also includes genes for FeoA, a small cytosolic protein likely to activate FeoB, and FeoC, a proposed transcriptional regulator [48]. There are two FeoB-encoding operons in *A. vinelandii*. One contains the FeoABC genes and the other only FeoAB genes. While no FeoC is found in the latter, it encodes a FeoB-associated Cys-rich membrane protein of unknown function that is also present in other species lacking FeoC [49]. Mutations in *feoB* genes often result in iron deficiency, and these mutants require iron supplementation of the culture medium to grow [50,51]. However, no study on FeoB proteins in *A. vinelandii* has been published to date. In addition to the Feo proteins, the *A. vinelandii* genome encodes other putative iron uptake proteins of the ZIP or NRAMP families. Members of these families have been shown to participate in iron uptake from soil in other organisms [52,53,54,55], but their role in *A. vinelandii* has not been determined. Studies using single, double, and multiple mutants are needed to determine the relative importance of each transport system and the specific environmental conditions for which they were selected.

## 3. Iron Trafficking in the Cytosol

Iron is recovered from siderophores in the periplasm or cytosol by the reduction or degradation of the chelator (Figure 2). Broad-spectrum ferric siderophore reductases use the electrons provided by NADH to release iron [56]. In *A. vinelandii*, cytosolic ferric reductase activity has been identified in the cytosol. This activity reduces iron provided as a complex with azotochelin and, with a lower activity, azotobactin [57]. Enzymes that perform this function in bacteria include Fre reductase or FhuF proteins [58,59]. The *A. vinelandii* genome encodes an orthologue of the former enzyme, which would likely be responsible for reductive iron recovery from siderophores. Alternatively, iron could be released by hydrolyzing the chelator. Although not many of these enzymes have been identified, several esterases (such as Fes esterase or IroD or IroE) have been proposed to release iron from catecholate chelators [60]. *A. vinelandii* encodes two putative members of the IroE family in proximity to genes involved in siderophore–iron uptake. 

Once in the cytosol, iron cannot be free, in its hydrated form, but it must remain bound to soluble chelators or proteins. This is to prevent the non-enzymatic production of free radicals in Fenton-type reactions and to prevent mis-metallation of other proteins [3]. As a result, iron importers would not merely release iron into the cytosol, but they would transfer it to an acceptor molecule, and through the physical interaction of donor and acceptor molecules, iron would reach its final acceptor proteins. Intracellular amino acids or short peptides, such as glutathione, may serve as iron acceptors, acting as iron buffers [61,62,63]. However, given the large number of ferroproteins in a cell and their relatively similar iron affinity constants, we must look for additional, larger molecules that would act as iron chaperones. These molecules would not only act as iron carriers, but also by being able to dock with some proteins and not with others, they would add another layer of specificity to iron exchange beyond simple metal affinity constants. This is the case in mammalian cells, where the existence of specific iron chaperones has been described [64,65]. However, no such proteins have yet been isolated in bacteria. To identify them, we must characterize the proteins interacting with the known iron transporters, as well as those with known iron utilization nodes, in particular bacterioferritins/ferritins, Fe-S cluster scaffold proteins, and ferrochelatases. 

Ferritins are multimeric iron storage proteins present in all domains of life [66]. Typically, 24 monomers form a cage-like structure in which iron is stored [67] (Figure 2). Bacterial genomes can encode different classes of ferritins: Ftns, similar to animal ferritins; Bfrs, heme-containing bacterioferritins (Bfrs); and mini-ferritins (Dpn), with only 12 monomers instead of the typical 24 [68,69]. The genome of *A. vinelandii* encodes one ferritin, two bacterioferritins, and one mini-ferritin. Their functional role seems to vary from organism to organism. In *E. coli,* ferritins are important for adaptation from iron-sufficient to iron-deficient conditions, suggesting a role in iron storage and mobilization, while bacterioferritin mutants show no significant phenotype [70]. In *Salmonella enterica*, Bfrs are the main iron storage proteins [71]. Other proposed roles for these proteins include protection against O_2_ toxicity, tolerance to free radicals, and virulence [51,72]. Alternatively, mixed ferritins have been proposed in which the cage-like structure is formed by ferritin and bacterioferritin in an heteromer [73]. No experimental evidence for the role of these proteins in *A. vinelandii* has been provided to date.

The Fe-S clusters are assembled by the sequential addition of iron and sulfur to scaffold proteins and are used for essential cellular processes such as the tricarboxylic acid cycle or energy transduction [74]. Typically, IscU serves as the scaffold protein in most organisms, interacting with the cysteine desulfurase IscS to receive sulfur and with a yet-to-be determined protein that would provide iron (Figure 2). In this scaffold protein, a [Fe_2_S_2_] cluster is first assembled, which is later condensed into a [Fe_4_S_4_] cluster [75]. The required electrons are provided by a [Fe_2_S_2_] cluster-containing ferredoxin [76,77,78]. From IscU, the [Fe_4_S_4_] cluster is transferred to different iron carriers, which could include Nfu or IscA [79,80,81]. IscA has also been proposed to act as scaffold protein for [Fe_2_S_2_] cluster biosynthesis, although it appears to be essential only under high O_2_ conditions [82]. Glutaredoxins may also act as Fe-S cluster carrier proteins [83]. An additional Fe-S scaffold protein called NifU is found in *A. vinelandii* (Figure 2). In this protein, the activities of IscU, ferredoxin, and NfU have been combined as domains of the same polypeptide. NifU is the primary assembly point for the Fe-S clusters required for nitrogenase activity [79,84,85,86,87], whereas IscU has a housekeeping role [88]. This is evidenced by the lethal phenotype of *A. vinelandii iscU* mutants, while those affected in *nifU* have a reduced growth only under diazotrophic conditions, suggesting that IscU could partially replace NifU [89,90]. This differential role is not a consequence of the activity of each protein, but rather of their regulation since the overexpression of NifU could also revert the *iscU* phenotype. However, the IscS and NifS desulfurases are not functionally interchangeable [88]. Other proteins, such as NafF, may also perform Fe-S cluster carrier functions under diazotrophic conditions [77,82,90]. While many bacteria also produce Suf proteins, an additional Fe-S cluster biosynthetic system where expression is often regulated by stress conditions [91,92], no *suf* gene can be found in the *A. vinelandii* genome sequence.

Finally, the genome of *A. vinelandii* contains one ferrochelatase gen, HemH [93]. Ferrochelatases are responsible for iron insertion into protoporphyrin IX during heme group biosynthesis [94,95].

## 4. Iron Transport during Nitrogen Fixation

As mentioned above, *A. vinelandii* can grow on N_2_ as the sole nitrogen source using the nitrogenase enzyme [17,18]. *A. vinelandii* carries the three known classes of nitrogenase: the Mo nitrogenase [96], the V nitrogenase [97], and the Fe-only nitrogenase [98]. All nitrogenases require metal clusters to function: an [Fe_4_S_4_] cluster at the interface of the dimeric dinitrogenase reductase (NifH in the Mo nitrogenase), the [Fe_8_S_7_] P-cluster located at the interface of each αβ pair of subunits of the dinitrogenase component (NifDK in the Mo nitrogenase), and the FeMo-co/FeV-co/FeFe-co (Fe_7_S_9_M-C-homocitrate; M being Mo, V or Fe, respectively) embedded in each α subunit of the dinitrogenase component (NifD in the Mo nitrogenase) [97,99,100,101]. The biosynthesis of these cofactors and their high iron requirements have recently been reviewed [100,102,103]. It has been estimated that *A. vinelandii* cells expressing nitrogenase contain up to 125 μM of NifH and 50 μM of NifDK, implying that 2 mM iron is solely dedicated to nitrogenase function [104]. Therefore, *A. vinelandii* requires an efficient mechanism of iron uptake. This is in contrast to what occurs during symbiotic nitrogen fixation, as the diazotroph will receive the required iron from the host through dedicated nodule metal transporters [105,106]. 

**Figure 3:**
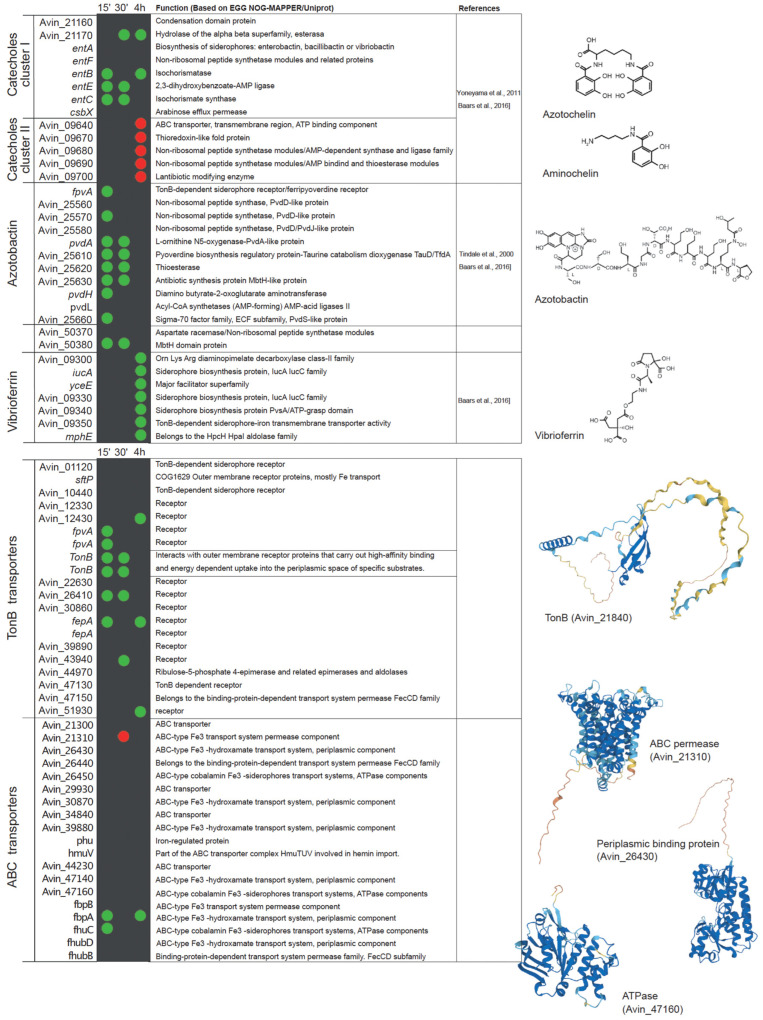

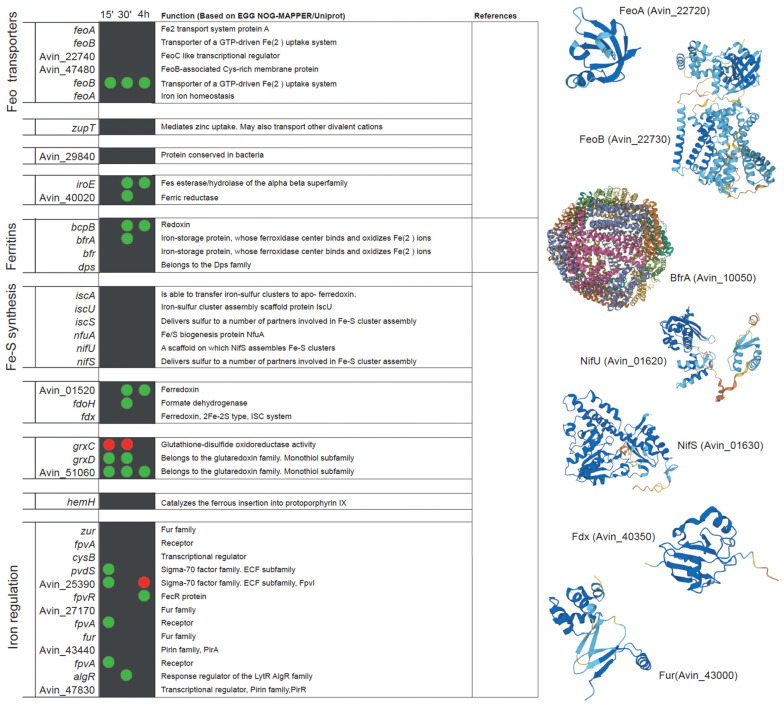
List of *A. vinelandii* genes involved or putatively involved in iron homeostasis and their regulation under diazotrophic conditions. Genes are organized by function and named according to their accession numbers in the published genome [93,103]. Green and red dots indicate the up-regulation or down-regulation, respectively, of the expression of each gene in nitrogenase de-repressing conditions compared to nitrogen-sufficient conditions (NH_3_) at three time points after the removal of NH_3_ from the medium (15 min, 30 min, and 4 h). No dot means that no change in expression was observed. This comparison was made using the transcriptomic data deposited in Gene Expression Ommibus Accesion GSE244772. Functional annotations were obtained from EGG NOG-MAPPER and Uniprot. Structural models were generated using AlphaFold [109] and visualized with PyMOL (Schörindger, Inc, New York, NY, USA). The references indicated are Baars et al., 2016 [31], Yoneyama et al., 2011 [110], and Tindale et al., 2021 [111].

Analysis of publicly available transcriptional datasets of the early stages of nitrogenase de-repression supports this statement [96]. Within fifteen minutes after the removal of ammonium from the medium, *A. vinelandii* induces the expression of genes involved in iron uptake, including Fe^2+^ and siderophore-bound forms (Figure 3). The role of FeoAB transport systems in iron uptake during nitrogen fixation has also been reported in *Bradyrhizobium japonicum,* where the mutation of *feoA* or *feoB* results in the loss of nitrogenase activity in nodules [107]. Similarly, siderophore and iron ABC transporters can be found to be up-regulated in *Medicago truncatula* nodules colonized by *Sinorhizobium meliloti* [105]. The catechol siderophore and azotobactin biosynthetic pathways are also up-regulated in *A. vinelandii* during diazotrophic conditions, as is the expression of a bacterioferritin in an attempt to scavenge as much iron as possible. To ensure the recovery of iron siderophores, a number of TonB-like proteins are also up-regulated. However, there seems to be some specificity, as other proteins of the same family putatively involved in iron uptake are down-regulated (Figure 3). Similarly, ABC transporters related to iron uptake are also induced (Figure 3). During this short period, the expression of two glutaredoxins is increased, suggesting a role in adaptation to diazotrophy. This is consistent with the reduction in nitrogenase activity of *Medicago* nodules inoculated with strains mutated in glutaredoxins [108]. Within 30 min of de-repression, enzymes involved in iron recovery from siderophores are up-regulated (Figure 3). Iron storage in bacterioferritins still seems to be important as indicated by the up-regulation of *BfrA* and *Dps*. Ferredoxins are also more highly expressed, which could be the result of enhanced electron requirement for nitrogen fixation. Catechol siderophores synthesized by cluster 2 (Figure 1C) seem to be less important at this time point, and consequently, their expression is reduced (Figure 3). After 4 h of de-repression, *A. vinelandii* induces the expression of genes involved in the vibrioferrin siderophore synthesis pathway (Figure 3), and still maintains the high expression levels of the cluster 1 of the cathechol siderophore pathway. However, cluster 2 is down-regulated, and no significant differences in the expression of genes in the azobactin synthesis pathways can be found between diazotrophically grown *A. vinelandii* or when they are grown with ammonia. Similarly, an esterase is still induced, probably to recover iron from siderophores. Two TonB-like transporters and two ABC transporters putatively involved in iron uptake are still up-regulated at this time point (Figure 3).

## 5. Regulation of Iron Homeostasis

Not much is known about the mechanism(s) that senses cytosolic iron in *A. vinelandii*. The iron-sensing system involving the ferric uptake regulator protein (Fur) is widely distributed in the bacterial phylogeny [112]. Fur regulates the iron metabolism [113,114,115,116], by binding to a 19 pb DNA (Fur box) that prevents the access of RNA polymerase, resulting in the repression of downstream genes [117]. Although *A. vinelandii* has two Fur homologues (Figure 3), it is not known how iron uptake is increased under diazotrophic conditions, whether it is simply a response to the decreasing cytosolic iron levels or a part of a more complex system coupled to the de-represssion of *nif* genes. 

Iron transport can be indirectly regulated by two extra cytoplasmic function sigma factors (ECFs): PvdS and FpvI [42]. The genome of *A. vinelandii* contains two PvdS-like genes and one FpvI. PvdS and FpvI factors are sequestered by FpvR, a protein located in the inner membrane. When Fe^3+^ is bound to PVD, a Fe–PVD complex is formed, and when transported by FpvA, it causes the degradation of FpvR and the release of PvdS and FpvI factors [118]. There are three candidate *fpvA* genes in *A. vinelandii*. Interestingly, two of these genes are up-regulated in the early stage of adaptation to diazotrophic growth conditions (Figure 3). This would trigger the synthesis of siderophores to increase iron uptake for nitrogenase synthesis. However, at 30 min of adaptation, the expression levels are similar to those of *A. vinelandii* grown with ammonia. Moreover, the induction of *fpvR* at 4 h could indicate a reduction in pyoverdine synthesis, also supported by the down-regulation of *fpvI* (Figure 3). 

In *P. aeruginosa,* siderophore biosynthesis can also be controlled by the phosphorylation state of AlgR. In the non-phosphorylated state, AlgR decreases the pyoverdine production, whereas it increases it when phosphorylated [119]. The *A. vinelandii* AlgR orthologue is also up-regulated 30 min after its transfer to diazotrophic conditions, suggesting a role in increasing iron uptake for nitrogen fixation (Figure 3).

## 6. Conclusions

In recent years, there has been renewed interest in nitrogen fixation and its biochemistry. While considerable progress has been made in understanding how the nitrogenase enzyme works [17] and how its metal cofactors are synthesized [100], much less is known about how iron is supplied and how diazotrophic bacteria control iron homeostasis. In this review, we have outlined what is known, and what can be inferred from other bacterial systems or from transcriptomic data. However, much experimental work is needed to determine the relative importance of the large diversity of iron import systems in *A. vinelandii*, the mechanisms of intracellular iron allocation, and how iron homeostasis is controlled in diazotrophy. All of this information will be valuable not only for understanding a key biochemical process in the biosphere but also for translating it to crops engineered to produce active nitrogenase [21,120].

## Data Availability

All the data used in this manuscript are available upon request. Transcriptional data for Figure 3 were obtained from Gene Expression Ommibus Accesion GSE244772.

## References

[1] Bandyopadhyay S., Chandramouli K., Johnson M.K. (2008). Iron-sulfur cluster biosynthesis. Biochem. Soc. Trans..

[2] Layer G. (2021). Heme biosynthesis in prokaryotes. Biochim. Biophys. Acta Mol. Cell Res..

[3] Winterbourn C.C. (1995). Toxicity of iron and hydrogen peroxide: The Fenton reaction. Toxicol. Lett..

[4] Stanbury D.M. (2022). The principle of detailed balancing, the iron-catalyzed disproportionation of hydrogen peroxide, and the fenton reaction. Dalton Trans..

[5] Tang D., Chen X., Kang R., Kroemer G. (2021). Ferroptosis: Molecular mechanisms and health implications. Cell Res..

[6] Checa-Fernandez A., Santos A., Romero A., Dominguez C.M. (2021). Application of chelating agents to enhance fenton process in soil remediation: A review. Catalysts.

[7] Murgia I., Marzorati F., Vigani G., Morandini P. (2022). Plant iron nutrition: The long road from soil to seeds. J. Exp. Bot..

[8] Ikeda-Ohtsubo W., Brugman S., Warden C.H., Rebel J.M.J., Folkerts G., Pieterse C.M.J. (2018). How can we define “optimal microbiota?”: A comparative review of structure and functions of microbiota of animals, fish, and plants in agriculture. Front. Nutr..

[9] Harbort C.J., Hashimoto M., Inoue H., Niu Y., Guan R., Rombolà A.D., Kopriva S., Voges M.J.E.E.E., Sattely E.S., Garrido-Oter R. (2020). Root-secreted coumarins and the microbiota interact to improve iron nutrition in *Arabidopsis*. Cell Host Microbe.

[10] Andrews S., Norton I., Salunkhe A.S., Goodluck H., Aly W.S.M., Mourad-Agha H., Cornelis P. (2013). Control of iron metabolism in bacteria. Met. Ions Life Sci..

[11] Hantke K. (2001). Iron and metal regulation in bacteria. Curr. Opin. Microbiol..

[12] Semsey S., Andersson A.M.C., Krishna S., Jensen M.H., Massé E., Sneppen K. (2006). Genetic regulation of fluxes: Iron homeostasis of *Escherichia coli*. Nucleic Acids Res..

[13] Trindade I.B., Silva J.M., Fonseca B.M., Catarino T., Fujita M., Matias P.M., Moe E., Louro R.O. (2019). Structure and reactivity of a siderophore-interacting protein from the marine bacterium *Shewanella* reveals unanticipated functional versatility. J. Biol. Chem..

[14] Schalk I.J., Perraud Q. (2023). *Pseudomonas aeruginosa* and its multiple strategies to access iron. Environ. Microbiol..

[15] Grass G. (2006). Iron transport in *Escherichia coli*: All has not been said and done. BioMetals.

[16] O’Brian M.R. (2015). Perception and homeostatic control of iron in the Rhizobia and related bacteria. Annu. Rev. Microbiol..

[17] Martin del Campo J.S., Rigsbee J., Bueno Batista M., Mus F., Rubio L.M., Einsle O., Peters J.W., Dixon R., Dean D.R., Dos Santos P.C. (2022). Overview of physiological, biochemical, and regulatory aspects of nitrogen fixation in *Azotobacter vinelandii*. Crit. Rev. Biochem. Mol. Biol..

[18] Hoffman B.M., Lukoyanov D., Yang Z.Y., Dean D.R., Seefeldt L.C. (2014). Mechanism of nitrogen fixation by nitrogenase: The next Stage. Chem. Rev..

[19] Aquilanti L., Favilli F., Clementi F. (2004). Comparison of different strategies for isolation and preliminary identification of Azotobacter from soil samples. Soil. Biol. Biochem..

[20] Alleman A.B., Mus F., Peters J.W. (2021). Metabolic model of the nitrogen-fixing obligate aerobe *Azotobacter vinelandii* predicts its adaptation to oxygen concentration and metal availability. Mbio.

[21] Burén S., Rubio L.M. (2018). State of the art in eukaryotic nitrogenase engineering. FEMS Microbiol. Lett..

[22] Kasa P., Modugapalem H., Battini K. (2015). Isolation, screening, and molecular characterization of plant growth promoting rhizobacteria isolates of Azotobacter and Trichoderma and their beneficial activities. J. Nat. Sci. Biol. Med..

[23] Nosheen A., Bano A., Yasmin H., Keyani R., Habib R., Shah S.T.A., Naz R. (2016). Protein quantity and quality of safflower seed improved by NP fertilizer and rhizobacteria (*Azospirillum* and *Azotobacter* Spp.). Front. Plant Sci..

[24] Bhosale H.J., Kadam T.A., Bobade A.R. (2013). Identification and production of *Azotobacter vinelandii* and its antifungal activity against *Fusarium oxysporum*. J. Environ. Biol..

[25] Frey P.A., Reed G.H. (2012). The ubiquity of iron. ACS Chem. Biol..

[26] Jones J.D. (2020). Iron availability and management considerations: A 4R approach. Crops Soils.

[27] Piskin E., Cianciosi D., Gulec S., Tomas M., Capanoglu E. (2022). Iron absorption: Factors, limitations, and improvement methods. ACS Omega.

[28] Kraepiel A.M.L., Bellenger J.P., Wichard T., Morel F.M.M. (2009). Multiple roles of siderophores in free-living nitrogen-fixing bacteria. BioMetals.

[29] Kramer J., Özkaya Ö., Kümmerli R. (2020). Bacterial siderophores in community and host interactions. Nat. Rev. Microbiol..

[30] Palanché T., Blanc S., Hennard C., Abdallah M.A., Albrecht-Gary A.M. (2004). Bacterial iron transport: Coordination properties of azotobactin, the highly fluorescent siderophore of *Azotobacter vinelandii*. Inorg. Chem..

[31] Baars O., Zhang X., Morel F.M.M., Seyedsayamdost M.R. (2016). The siderophore metabolome of *Azotobacter vinelandii*. Appl. Environ. Microbiol..

[32] Srivastava S., Dong H., Baars O., Sheng Y. (2023). Bioavailability of mineral-associated trace metals as cofactors for nitrogen fixation by *Azotobacter vinelandii*. Geobiology.

[33] McRose D.L., Baars O., Morel F.M.M., Kraepiel A.M.L. (2017). Siderophore production in *Azotobacter vinelandii* in response to Fe-, Mo- and V-limitation. Environ. Microbiol..

[34] Noar J.D., Bruno-Bárcena J.M. (2018). *Azotobacter vinelandii*: The source of 100 years of discoveries and many more to come. Microbiology.

[35] Thomas W., Bellenger J.P., Morel F.M.M., Kraepiel A.M.L. (2009). Role of the siderophore azotobactin in the bacterial acquisition of nitrogenase metal cofactors. Environ. Sci. Technol..

[36] Lange M.D., Abernathy J., Shoemaker C.A., Zhang D., Kirby A., Peatman E., Beck B.H. (2020). Proteome analysis of virulent *Aeromonas hydrophila* reveals the upregulation of iron acquisition systems in the presence of a xenosiderophore. FEMS Microbiol. Lett..

[37] Rizvi A., Khan M.S. (2018). Heavy metal induced oxidative damage and root morphology alterations of maize (*Zea Mays* L.) Plants and stress mitigation by metal tolerant nitrogen fixing *Azotobacter chroococcum*. Ecotoxicol. Environ. Saf..

[38] Quistgaard E.M., Löw C., Guettou F., Nordlund P. (2016). Understanding transport by the major facilitator superfamily (MFS): Structures pave the way. Nat. Rev. Mol. Cell Biol..

[39] Nikaido H. (1996). Multidrug efflux pumps of gram-negative bacteria. J. Bacteriol..

[40] Page W.J., Kwon E., Cornish A.S., Tindale A.E. (2003). The CsbX Gene of *Azotobacter vinelandii* encodes an MFS efflux pump required for catecholate siderophore export. FEMS Microbiol. Lett..

[41] Poole K., Krebes K., Mcnally C., Neshat S. (1993). Multiple antibiotic resistance in *Pseudomonas aeruginosa: Evidence* for involvement of an efflux operon. J. Bacteriol..

[42] Visca P., Imperi F., Lamont I.L. (2007). Pyoverdine siderophores: From biogenesis to biosignificance. Trends Microbiol..

[43] Lim B.L. (2010). TonB-dependent receptors in nitrogen-fixing nodulating bacteria. Microbes Environ..

[44] Ferguson A.D., Deisenhofer J. (2002). TonB-dependent receptors-structural perspectives. Biochim. Biophys. Acta Biomembr..

[45] Thomas C., Tampé R. (2020). Structural and mechanistic principles of ABC transporters. Annu. Rev. Biochem..

[46] Altendorf K., Stalz W.D., Greie J.C., Deckers-Hebestreit G. (2000). Structure and function of the F_o_ complex of the ATP synthase from *Escherichia coli*. J. Exp. Biol..

[47] Ash M.R., Maher M.J., Guss J.M., Jormakka M. (2011). The initiation of GTP hydrolysis by the G-domain of FeoB: Insights from a transition-state complex structure. PLoS ONE.

[48] Shin M., Park J., Jin Y., Kim I.J., Payne S.M., Kim K.H. (2020). Biochemical characterization of bacterial FeoBs: A perspective on nucleotide specificity. Arch. Biochem. Biophys..

[49] Lau C.K.Y., Krewulak K.D., Vogel H.J. (2016). Bacterial ferrous iron transport: The feo system. FEMS Microbiol. Rev..

[50] Robey M., Cianciotto N.P. (2002). *Legionella pneumophila* FeoAB promotes ferrous iron uptake and intracellular infection. Infect. Immun..

[51] Velayudhan J., Hughes N.J., McColm A.A., Bagshaw J., Clayton C.L., Andrews S.C., Kelly D.J. (2000). Iron acquisition and virulence in *Helicobacter pylori*: A major role for FeoB, a high-affinity ferrous iron transporter. Mol. Microbiol..

[52] Bozzi A.T., Gaudet R. (2021). Molecular mechanism of Nramp-family transition metal transport. J. Mol. Biol..

[53] Grass G., Franke S., Taudte N., Nies D.H., Kucharski L.M., Maguire M.E., Rensing C. (2005). The metal permease ZupT from *Escherichia coli* is a transporter with a broad substrate spectrum. J. Bacteriol..

[54] Makui H., Roig E., Cole S.T., Helmann J.D., Gros P., Cellier M.F.M. (2000). Identification of the *Escherichia coli* K-12 Nramp orthologue (MntH) as a selective divalent metal ion transporter. Mol. Microbiol..

[55] Roberts C.S., Ni F., Mitra B. (2021). The zinc and iron binuclear transport center of ZupT, a ZIP transporter from *Escherichia coli*. Biochemistry.

[56] Fischer E., Strehlow B., Hartz D., Braun V. (1990). Soluble and membrane-bound ferrisiderophore reductases of *Escherichia coli* K-12. Arch. Microbiol..

[57] Huyer M., Page W.J. (1989). Ferric reductase activity in *Azotobacter vinelandii* and its inhibition by Zn^2+^. J. Bacteriol..

[58] Cain T.J., Smith A.T. (2021). Ferric iron reductases and their contribution to unicellular ferrous iron uptake. J. Inorg. Biochem..

[59] Müller K., Matzanke B.F., Schünemann V., Trautwein A.X., Hantke K. (1998). FhuF, an iron-regulated protein of *Escherichia coli* with a new type of [2Fe-2S] center. Eur. J. Biochem..

[60] Lin H., Fischbach M.A., Liu D.R., Walsh C.T. (2005). In vitro characterization of salmochelin and enterobactin trilactone hydrolases IroD, IroE, and Fes. J. Am. Chem. Soc..

[61] Brissot P., Ropert M., Le Lan C., Loréal O. (2012). Non-transferrin bound iron: A key role in iron overload and iron toxicity. Biochim. Biophys. Acta Gen. Subj..

[62] Galaris D., Barbouti A., Pantopoulos K. (2019). Iron homeostasis and oxidative stress: An intimate relationship. Biochim. Biophys. Acta Mol. Cell Res..

[63] Hider R., Aviles M.V., Chen Y.L., Latunde-Dada G.O. (2021). The role of Gsh in intracellular iron trafficking. Int. J. Mol. Sci..

[64] Yanatori I., Richardson D.R., Imada K., Kishi F. (2016). Iron export through the transporter ferroportin 1 is modulated by the iron chaperone PCBP2. J. Biol. Chem..

[65] Philpott C.C., Ryu M.S., Frey A., Patel S. (2017). Cytosolic iron chaperones: Proteins delivering iron cofactors in the cytosol of mammalian cells. J. Biol. Chem..

[66] Arosio P., Elia L., Poli M. (2017). Ferritin, cellular iron storage and regulation. IUBMB Life.

[67] Gijsbers A., Zhang Y., Gao Y., Peters P.J., Ravelli R.B.G. (2021). *Mycobacterium tuberculosis* ferritin: A suitable workhorse protein for cryo-EM development. Acta Crystallogr. D Struct. Biol..

[68] Guo M., Gao M., Liu J., Xu N., Wang H. (2022). Bacterioferritin nanocage: Structure, biological function, catalytic mechanism, self-assembly and potential applications. Biotechnol. Adv..

[69] Liu X., Kim K., Leighton T., Theil E.C. (2006). Paired *Bacillus anthracis* Dps (mini-ferritin) have different reactivities with peroxide. J. Biol. Chem..

[70] Abdul-tehrani H., Hudson A.J., Chang Y., Timms A.R., Hawkins C., Williams J.M., Harrison P.M., Guest J.R., Andrews S.C. (1999). Ferritin mutants of *Escherichia coli* are iron deficient and growth impaired, and Fur mutants are iron deficient. J. Bacteriol..

[71] Velayudhan J., Castor M., Richardson A., Main-Hester K.L., Fang F.C. (2007). The role of ferritins in the physiology of *Salmonella enterica* Sv. Typhimurium: A unique role for ferritin B in iron-sulphur cluster repair and virulence. Mol. Microbiol..

[72] Ceci P., Ilari A., Falvo E., Chiancone E. (2003). The Dps protein of *Agrobacterium tumefaciens* does not bind to DNA but protects it toward oxidative cleavage. X-ray crystal structure, iron binding, and hydroxyl-radical scavenging properties. J. Biol. Chem..

[73] Yao H., Soldano A., Fontenot L., Donnarumma F., Lovell S., Chandler J.R., Rivera M. (2022). *Pseudomonas aeruginosa* bacterioferritin is assembled from FtnA and BfrB subunits with the relative proportions dependent on the environmental oxygen availability. Biomolecules.

[74] Lill R., Freibert S.-A. (2020). Mechanisms of mitochondrial iron-sulfur protein biogenesis. Annu. Rev. Biochem..

[75] Agar J.N., Krebs C., Frazzon J., Huynh B.H., Dean D.R., Johnson M.K. (2000). IscU as a scaffold for iron-sulfur cluster biosynthesis: Sequential assembly of [2Fe-2S] and [4Fe-4S] clusters in IscU. Biochemistry.

[76] Ollagnier-De-Choudens S., Mattioli T., Takahashi Y., Fontecave M. (2001). Iron-sulfur cluster assembly. Characterization of IscA and evidence for a specific and functional complex with ferredoxin. J. Biol. Chem..

[77] Zheng L., Cash V.L., Flint D.H., Dean D.R. (1998). Assembly of iron-sulfur clusters: Identification of an iscSUA-hscBA-fdx gene cluster from *Azotobacter vinelandii*. J. Biol. Chem..

[78] Yoch D.C., Arnon D.I. (1975). Comparison of two ferredoxins from *Rhodospirillum rubrum* as electron carriers for the native nitrogenase. J. Biol. Chem..

[79] Bandyopadhyay S., Naik S.G., O’Carroll I.P., Huynh B.H., Dean D.R., Johnson M.K., Dos Santos P.C. (2008). A proposed role for the *Azotobacter vinelandii* Nfua protein as an intermediate iron-sulfur cluster carrier. J. Biol. Chem..

[80] Cai K., Frederick R.O., Markley J.L. (2020). ISCU interacts with NFU1, and ISCU [4Fe-4S] transfers its Fe-S cluster to NFU1 leading to the production of holo-NFU1. J. Struct. Biol..

[81] Ding B., Smith E.S., Ding H. (2005). Mobilization of the iron centre in IscA for the iron-sulphur cluster assembly in IscU. Biochem. J..

[82] Krebs C., Agar J.N., Smith A.D., Frazzon J., Dean D.R., Huynh B.H., Johnson M.K. (2001). IscA, an alternate scaffold for Fe-S cluster biosynthesis. Biochemistry.

[83] Braymer J.J., Freibert S.A., Rakwalska-Bange M., Lill R. (2021). Mechanistic concepts of iron-sulfur protein biogenesis in biology. Biochim. Biophys. Acta Mol. Cell Res..

[84] Smith A.D., Jameson G.N.L., Dos Santos P.C., Agar J.N., Naik S., Krebs C., Frazzon J., Dean D.R., Huynh B.H., Johnson M.K. (2005). NifS-mediated assembly of [4Fe-4S] clusters in the N- and C-terminal domains of the NifU scaffold protein. Biochemistry.

[85] Agar J.N., Yuvaniyama P., Jack R.F., Cash V.L., Smith A.D., Dean D.R., Johnson M.K. (2000). Modular organization and identification of a mononuclear iron-binding site within the NifU Protein. J. Biol. Inorg. Chem..

[86] Dos Santos P.C., Smith A.D., Frazzon J., Cash V.L., Johnson M.K., Dean D.R. (2004). Iron-sulfur cluster assembly: NifU-directed activation of the nitrogenase Fe protein. J. Biol. Chem..

[87] Fu W., Jack R.F., Morgan T.V., Dean D.R., Johnson M.K. (1994). NifU gene product from *Azotobacter vinelandii* is a homodimer that contains two identical [2Fe-2S] clusters. Biochemistry.

[88] Dos Santos P.C., Johnson D.C., Ragle B.E., Unciuleac M.C., Dean D.R. (2007). Controlled expression of Nif and Isc iron-sulfur protein maturation components reveals target specificity and limited functional replacement between the two systems. J. Bacteriol..

[89] Johnson D.C., Unciuleac M.C., Dean D.R. (2006). Controlled expression and functional analysis of iron-sulfur cluster biosynthetic components within *Azotobacter vinelandii*. J. Bacteriol..

[90] Jaeobson M.R., Cash V.L., Weiss M.C., Laird N.F., Newton W.E., Dean D.R. (1989). Biochemical and genetic analysis of the nifUSVWZM Cluster from *Azotobacter vinelandii*. Mol. Genet. Genom..

[91] Sendra M., Ollagnier de Choudens S., Lascoux D., Sanakis Y., Fontecave M. (2007). The SUF iron-sulfur cluster biosynthetic machinery: Sulfur transfer from the SUFS-SUFE complex to SUFA. FEBS Lett..

[92] Bai Y., Chen T., Happe T., Lu Y., Sawyer A. (2018). Iron-sulphur cluster biogenesis via the SUF pathway. Metallomics.

[93] Setubal J.C., Dos Santos P., Goldman B.S., Ertesvåg H., Espin G., Rubio L.M., Valla S., Almeida N.F., Balasubramanian D., Cromes L. (2009). Genome sequence of *Azotobacter vinelandii*, an obligate aerobe specialized to support diverse anaerobic metabolic processes. J. Bacteriol..

[94] Ferreira G.C. (1999). Ferrochelatase. Int. J. Biochem. Cell Biol..

[95] Obi C.D., Bhuiyan T., Dailey H.A., Medlock A.E. (2022). Ferrochelatase: Mapping the intersection of iron and porphyrin metabolism in the mitochondria. Front. Cell Dev. Biol..

[96] Rubio L.M., Ludden P.W. (2005). Maturation of nitrogenase: A biochemical puzzle. J. Bacteriol..

[97] Chisnell J.R., Premakumar R., Bishop P.E. (1988). Purification of a second alternative nitrogenase from a NifHDK deletion strain of *Azotobacter vinelandii*. J. Bacteriol..

[98] Rees J.A., Bjornsson R., Schlesier J., Sippel D., Einsle O., DeBeer S. (2015). The Fe–V cofactor of vanadium nitrogenase contains an interstitial carbon atom. Angew. Chem..

[99] Guo Y., Echavarri-Erasun C., Demuez M., Jiménez-Vicente E., Bominaar E.L., Rubio L.M. (2016). The nitrogenase FeMo-cofactor precursor formed by NifB protein: A diamagnetic cluster containing eight iron atoms. Angew. Chem..

[100] Burén S., Jiménez-Vicente E., Echavarri-Erasun C., Rubio L.M. (2020). Biosynthesis of Nitrogenase Cofactors. Chem. Rev..

[101] Hinnemann B., Nørskov J.K. (2004). Structure of the FeFe-cofactor of the iron-only nitrogenase and possible mechanism for dinitrogen reduction. Phys. Chem. Chem. Phys..

[102] Jasniewski A.J., Lee C.C., Ribbe M.W., Ribbe M.W., Hu Y. (2020). Reactivity, mechanism, and assembly of the alternative nitrogenases. Chem. Rev..

[103] Hu Y., Ribbe M.W. (2016). Biosynthesis of the metalloclusters of nitrogenases. Annu. Rev. Biochem..

[104] Poza-Carrión C., Jiménez-Vicente E., Navarro-Rodríguez M., Echavarri-Erasun C., Rubio L.M. (2014). Kinetics of Nif gene expression in a nitrogen-fixing bacterium. J. Bacteriol..

[105] Abreu I., Mihelj P., Raimunda D. (2019). Transition metal transporters in rhizobia: Tuning the inorganic micronutrient requirements to different living styles. Metallomics.

[106] González-Guerrero M., Navarro-Gómez C., Rosa-Núñez E., Echávarri-Erasun C., Imperial J., Escudero V. (2023). Forging a symbiosis: Transition metal delivery in symbiotic nitrogen fixation. New Phytol..

[107] Sankari S., O’Brian M.R. (2016). The *Bradyrhizobium japonicum* ferrous iron transporter FeoAB is required for ferric iron utilization in free living aerobic cells and for symbiosis. J. Biol. Chem..

[108] Benyamina S.M., Baldacci-Cresp F., Couturier J., Chibani K., Hopkins J., Bekki A., de Lajudie P., Rouhier N., Jacquot J.P., Alloing G. (2013). Two *Sinorhizobium meliloti* glutaredoxins regulate iron metabolism and symbiotic bacteroid differentiation. Environ. Microbiol..

[109] Jumper J., Evans R., Pritzel A., Green T., Figurnov M., Ronneberger O., Tunyasuvunakool K., Bates R., Žídek A., Potapenko A. (2021). Highly accurate protein structure prediction with AlphaFold. Nature.

[110] Yoneyama F., Yamamoto M., Hashimoto W., Murata K. (2011). *Azotobacter vinelandii* gene clusters for two types of peptidic and catechol siderophores produced in response to molybdenum. J. Appl. Microbiol..

[111] Tindale A.E., Mehrotra M., Ottem D., Page W.J. (2000). Dual regulation of catecholate siderophore biosynthesis in *Azotobacter vinelandii* by iron and oxidative stress. Microbiology.

[112] Steingard C.H., Helmann J.D. (2023). Meddling with metal sensors: Fur-family proteins as signaling hubs. J. Bacteriol..

[113] Huang M., Liu M., Liu J., Zhu D., Tang Q., Jia R., Chen S., Zhao X., Yang Q., Wu Y. (2021). Functional characterization of Fur in iron metabolism, oxidative stress resistance and virulence of *Riemerella anatipestifer*. Vet. Res..

[114] Seo S.W., Kim D., Latif H., O’Brien E.J., Szubin R., Palsson B.O. (2014). Deciphering Fur transcriptional regulatory network highlights its complex role beyond iron metabolism in *Escherichia coli*. Nat. Commun..

[115] Gao H., Ma L., Qin Q., Qiu Y., Zhang J., Li J., Lou J., Diao B., Zhao H., Shi Q. (2020). Fur represses *Vibrio cholerae* biofilm formation via direct regulation of VieSAB, CdgD, VpsU, and VpsA-K transcription. Front. Microbiol..

[116] Pinochet-Barros A., Helmanna J.D. (2020). *Bacillus subtilis* Fur is a transcriptional activator for the PerR-repressed pfeT gene, encoding an iron efflux pump. J. Bacteriol..

[117] Escolar L., Pérez-Martín J., Martín M., Ví V., De Lorenzo V. (1999). Opening the iron box: Transcriptional metalloregulation by the Fur protein. J. Bacteriol..

[118] Llamas M.A., Imperi F., Visca P., Lamont I.L. (2014). Cell-surface signaling in Pseudomonas: Stress responses, iron transport, and pathogenicity. FEMS Microbiol. Rev..

[119] Little A.S., Okkotsu Y., Reinhart A.A., Damron F.H., Barbier M., Barrett B., Oglesby-Sherrouse A.G., Goldberg J.B., Cody W.L., Schurr M.J. (2018). *Pseudomonas aeruginosa* Algr phosphorylation status differentially regulates pyocyanin and pyoverdine production. mBio.

[120] Oldroyd G.E.D., Dixon R. (2014). Biotechnological solutions to the nitrogen problem. Curr. Opin. Biotechnol..

